# Associations between polygenic risk for obesity, body mass index and cognitive performance: Age as moderating factor

**DOI:** 10.1016/j.bbih.2026.101321

**Published:** 2026-07-31

**Authors:** Mathilda Recht, Kathrin Finke, Marius Gruber, Andreas J. Forstner, Janik Goltermann, Maike Richter, Benjamin Straube, Elisabeth Schrammen, Moritz Rau, Jonathan Repple, Kira Flinkenflügel, Judith Krieger, Anna Kraus, Elisabeth J. Leehr, Tiana Borgers, Tilo Kircher, Udo Dannlowski, Nils Opel

**Affiliations:** a Institute for Translational Psychiatry, University of Münster, Münster, Germany; b Department of Psychiatry & Neuroscience, Campus Benjamin Franklin, Charité Universitätsmedizin Berlin, Berlin, Germany; c Department of Psychiatry and Psychotherapy, University of Marburg, Marburg, Germany; d Center for Mind, Brain and Behavior (CMBB), University of Marburg, Marburg, Germany; e Center for Human Genetics, University of Marburg, Marburg, Germany; f Institute of Human Genetics, University of Bonn, School of Medicine & University Hospital Bonn, Bonn, Germany; g Department of Psychiatry and Psychotherapy, Jena University Hospital, Jena, Germany; h Department of Neurology, Memory Center, Jena University Hospital, Jena, Germany; i Department of Psychiatry, Psychosomatic Medicine and Psychotherapy, Goethe University, Frankfurt, Germany; j Cooperative Brain Imaging Center - CoBIC, Goethe University Frankfurt, Frankfurt, Germany; k Institute of Neuroscience and Medicine (INM-1), Research Center Jülich, Jülich, Germany; l German Centre for Mental Health (DZPG), Site Berlin-Potsdam, Berlin, Germany; m Department of Psychiatry, Medical School and University Medical Center OWL, Protestant Hospital of the Bethel Foundation, Bielefeld University; n German Center for Mental Health (DZPG), Site Jena Magdeburg Halle, Germany; o Center for Intervention and Research on Adaptive and Maladaptive Brain Circuits Underlying Mental Health (C-I-R-C), Site Jena Magdeburg Halle, Germany

## Abstract

Obesity has been associated with cognitive impairment, yet the contribution of obesity-related genetic liability to cognition across the lifespan remains unclear.

Here, we investigated associations between body mass index (BMI), obesity polygenic risk score (PRS), and cognitive performance in 796 individuals from the FOR2107 Marburg-Münster Affective Disorders Cohort Study (MACS). Cognitive function was assessed using a neuropsychological test battery covering working memory, episodic memory, attention, processing speed, executive function, and crystallized intelligence.

Multivariate analyses of covariance revealed significant age-dependent associations of both BMI (ηp^2^(BMI*age) = 0.038, F (10,771) = 3.06, p = 0.001) and obesity PRS (ηp^2^(PRS*age) = 0.028, F (10,763) = 2.20, p = 0.016) with cognitive performance, indicating that these associations differed across lifespan. No significant sex moderation or overall multivariate associations of BMI or PRS with cognition were observed. Both interactions remained significant after adjustment for education, perceived stress, and childhood trauma. In a sensitivity analysis, the PRS*age interaction remained significant after additional adjustment for BMI. Follow-up linear regression analyses revealed that increasing age was associated with a more negative relationship of BMI with episodic memory (B = −0.087, SE = 0.029, p = 0.003) and a more positive relationship with crystallized intelligence (B = 0.089, SE = 0.032, p = 0.005). Similarly, increasing age was associated with a more negative relationship of obesity PRS with episodic memory (B = −0.083, SE = 0.031, p = 0.008) and a more positive relationship with working memory (B = 0.073, SE = 0.034, p = 0.032).

Ultimately, our findings suggest that age is a key moderator of the associations between BMI, PRS, and cognition. The persistence of the PRS*age interaction after adjustment for BMI indicates that obesity-related genetic liability may influence cognition through mechanisms beyond current body weight.

## 1 Introduction

The obesity epidemic constitutes one of the greatest public health threats of the 21st century. Affecting all socioeconomic and age groups worldwide (World Health Organization), the prevalence of obesity has nearly tripled since 1975, resulting in 13% of the global adult population being obese (BMI > 30 kg/m^2^) and 39% of the population being overweight (BMI > 25 kg/m^2^) in 2016 (World Health Organization, 2021). Obesity carries a substantial socioeconomic burden (Tremmel et al., 2017) and is a major risk factor for non-communicable diseases, including cardiovascular disease, type 2 diabetes mellitus, musculoskeletal disorders and certain types of cancer (Blüher, 2019). Furthermore, midlife obesity is one of the strongest risk factors for cognitive decline and significantly increases the likelihood of developing dementia (Dahl et al., 2005; Hassing et al., 2010; Forny-Germano et al., 2018).

Despite the undeniable influence of lifestyle factors such as physical inactivity, high fat diets, poor sleep quality, stress, and other environmental aspects, it is known that obesity arises from the interplay of both environmental and biological factors (Elks et al., 2012; Stunkard et al., 1990; Yang et al., 2015). Indeed, a growing body of literature has shown that approximately 40-70% of individual variability in BMI is attributable to genetic factors (Maes et al., 1997; Locke et al., 2015) and that this inherited risk strongly influences ones’ reaction to an obesogenic environment. The vast majority of BMI is not influenced by a monogenic mutation, but a result of a cumulative effect of many genetic variants. Although there may be a few exceptions, such as melanocortin-4 receptor deficiency (MC4R) (Farooqi et al., 2003) or variants at the fat mass and obesity associated locus on chromosome 16 (FTO) (Scuteri et al., 2007; Frayling et al., 2007), most research data strongly suggest a polygenic inheritance of obesity susceptibility (Hinney et al., 2010). Therefore, the polygenic risk score (PRS) for obesity, based on genome-wide association studies (GWAS), which summarises the estimated effects of 2.1 million common genetic variants, provides a powerful tool for quantifying inherited susceptibility to obesity (Khera et al., 2019).

Similar to the factors contributing to obesity, one's cognitive abilities show a remarkable degree of genetic inheritance (Plomin and Stumm, 2018; Genç et al., 2021). More specifically, not only can some of the variance in general cognitive status be attributed to genetic factors (Haworth et al., 2010), but many specific neurocognitive functions including working memory, attention and executive functions are thought to have genetic components (Mollon et al., 2021; Vogler et al., 2014; Knowles et al., 2014; Friedman et al., 2008). While there is evidence of certain individual alleles, such as the APOE-gene, that are linked to cognitive alterations such as the onset of Alzheimer's disease (Fortea et al., 2024), there is consensus that the general heritability of cognitive abilities is a polygenic phenomenon (Genç et al., 2021).

In addition, accumulating evidence suggests a partial overlap in the genetic architecture of obesity and cognitive performance (Marioni et al., 2016; Spieker et al., 2015; Sanchez-Roige et al., 2018; Lancaster et al., 2018). This raises the possibility that obesity-related genetic liability contributes directly to cognitive outcomes beyond its influence on body weight itself, suggesting that the relationship between obesity and cognition may be driven in part by mechanisms that are not fully modifiable through lifestyle interventions alone.

As a starting point, previous work by Marioni et al. found that polygenic risk for obesity accounted for a small (0.42%) but significant proportion of the variance in cognitive function (Marioni et al., 2016), and Xian et al. reported significant inverse associations between obesity PRS and several cognitive domains in a cohort of elderly, all-male, all-white twins (Xian et al., 2020).

However, it remains largely unclear whether these associations generalize across adulthood or vary as a function of age, sex, and psychosocial factors. To our knowledge, no previous study has systematically examined whether associations between obesity-related genetic liability and cognition vary with age or remain independent of current BMI.

Age represents a particularly relevant moderator candidate, as obesity-related metabolic, inflammatory, and vascular alterations accumulate over time and may increasingly affect cognitive functioning with advancing age (Nguyen et al., 2014; Buie et al., 2019). Furthermore, theoretical frameworks such as the resource-modulation hypothesis propose that age-related reductions in neural resources may amplify the phenotypic expression of genetic influences on cognition (Papenberg et al., 2015). Together, these considerations suggest that the associations between obesity, obesity-related genetic liability, and cognition may vary across the lifespan. Elucidating these questions is of considerable relevance for informing prevention strategies targeting cognitive decline.

The present study investigated associations between BMI, obesity PRS, and cognitive performance. Specifically, we hypothesized that age moderates the associations between BMI and cognition and between obesity PRS and cognition. We further tested whether age-dependent associations between obesity PRS and cognition remain significant after adjustment for BMI and the BMI*age interaction, which would suggest pathways linking obesity-related genetic liability to cognition beyond current body weight. Finally, cognitive domain-specific regression analyses were conducted to identify the cognitive domains underlying significant multivariate effects.

## 2 Materials and methods

### 2.1 Sample

For the present analyses we included all participants from the FOR2107 Marburg-Münster Affective Disorder Cohort Study (MACS) (Kircher et al., 2019), for whom complete data of their cognitive assessment, BMI, years of education, age, sex, and genetic information for PRS calculation were available. In addition, considering the complex confounding interrelations between BMI, cognition and psychopathology, we only included participants who did not suffer from present or lifetime psychiatric disorders according to a structured clinical interview as described in previous papers (Rau et al., 2026). MACS is a two-center research consortium studying the neurobiological foundations of affective disorders that recruited participants aged 18-65 years with West-European ancestry from January 09, 2015 to May 11, 2018 via newspaper advertisements or local psychiatric hospitals (Kircher et al., 2019; Vogelbacher et al., 2018) More details on inclusion and exclusion criteria of the study population can be found in the supplements. Study procedures were approved by the Ethics Committees of the University of Münster (2014-422-b-S) and Marburg (07/14) following the Declaration of Helsinki. All participants signed informed consent before participation and received financial compensation. These inclusion criteria were met by n = 796 participants, resulting in a study sample of n = 288 male and n = 508 female participants. Further characteristics of our study sample are laid out in Table 1.

**Table 1:** Descriptive statistics of the study sample.

Characteristics	Descriptive statistics			
	Mean (SD^a^)	Median (IQR^b^)	Range	Frequency n (%)
Age	34.41 (12.79)	30 (24-46)	18-65	
BMI^c^	24.17 (4.41)	23.23 (21.22-26.02)	16.61-59.52	
Underweight (<18.5 kg/m^2^)				20 (2.51)
Normal weight (18.5 – 24.9 kg/m^2^)				512 (64.32)
Overweight (25.0-29.9 kg/m^2^)				194 (24.37)
Obesity (≥30 kg/m^2^)				70 (8.79)
Years of Education	14.02 (2.57)	13 (13-16)	9-18	
**Sex**				
Male				288 (36.18)
Female				508 (63.82)

Note.

a SD = standard deviation.

b IQR = Interquartile Range.

c BMI = body mass index.

### 2.2 Genetic methods & polygenic risk score calculation

Following previously published protocols (Opel et al., 2020a), genome-wide genotyping was performed for every individual in our study sample (n = 796) using the Infinium PsychArray-24 BeadChip (Illumina, San Diego, CA, USA).

With the use of the PLINK software, quality control procedures were implemented.

The present study excluded two samples that exhibited low genotype rates (<95%), sex inconsistencies (X-chromosome heterozygosity), and genetically related individuals. Furthermore, we excluded SNPs with a low genotyping rate (<95%), strand ambiguity (A/T and C/G SNPs), a low minor allele frequency (MAF <1%), or that showed deviation from Hardy-Weinberg equilibrium (p < 10-6).

Genotype data underwent imputation using the Haplotype Reference Consortium (HRC) reference panel (version v3.20101123) via minimac (McCarthy et al., 2016). Following imputation, only autosomal SNPs meeting quality control thresholds (MAF > 0.1 and imputation quality score > 0.9) were retained. These variants were converted to best-guess genotypes and included in subsequent polygenic analyses. The major histocompatibility complex (MHC) region was excluded from polygenic score calculations due to its complex linkage disequilibrium structure.

We operationalize genetic liability to obesity as a polygenetic risk score (Khera et al., 2019).

PRSs were calculated based on summary statistics from a large genome-wide association study (GWAS) of BMI conducted by Locke et al. (2015). The sample comprised 339,224 individuals and was independent of the MACS sample included in our study. With the use of the PLINK software and based on a p-value threshold at 1.0. The p-value threshold of 1.0 was selected a priori to capture the highly polygenic architecture of obesity, as inclusion of a large number of variants has been shown to maximize predictive performance for highly polygenic traits. SNPs were clumped for linkage disequilibrium using r^2^ = 0.1 and a physical distance threshold of 250 kb (plink –clump-p1 1 –clump-p2 1 –clump-r2 0.1 –clump-kb 250) (Chang et al., 2015).

For polygenetic risk score derivation, the sum of reference SNP alleles for each participant was multiplied by its effect size derived from the GWAS statistics to factor in the strength of its association with obesity. Subsequently, higher polygenetic risk scores imply greater probability for obesity.

### 2.3 Neurocognitive assessment

To assess cognitive performance, a neurocognitive test battery was applied, providing data on the cognitive domains of working memory, executive functioning, processing speed, attention, verbal learning and memory and verbal intelligence. Thereafter, composite scores were created from the individual neurocognitive tests, with each composite representing a specific cognitive domain. Cognitive test scores were z-standardized prior to composite calculation.

**Working memory:** In order to evaluate the individual visuospatial working memory capacity, we used the Corsi Block-Tapping Task in a forward (Corsi A) and backward version (Corsi B) (Kessels et al., 2000). Additionally, the letter-number sequencing test (LNST) was used to measure the verbal working memory capacity (Wechsler, 1997).

**Episodic memory:** The verbal learning and memory test (Helmstaedter and Durwen, 1990), a German adaption of the Rey Auditory Verbal Learning test (Rey, 1964), was applied to assess declarative learning and memory of a list of verbal items (VLMT A) (Helmstaedter and Durwen, 1990). Delayed recall from long-term memory of that same material assessed was after 25-30 min (RichtigA7).

**Attention:** Selective and sustained attention was assessed with the D2-test (Brickenkamp et al., 2010).

**Crystallized intelligence:** We conducted the MWT-B assessment, a German multiple-choice vocabulary test (Lehrl, 2005) to assess verbal intelligence. The MWT-B score was directly converted into IQ points for the purpose of analysis.

**Processing speed:** Processing speed was evaluated with both the Trail-Making Test part A (TMT A) (Reitan, 1958; Sánchez-Cubillo et al., 2009) and the Digit Symbol Substitution Test (DSST) (Wechsler, 1997). TMT A was used as a measure of visuoperceptual processing speed. Test version TMT B entails switching between digits and letters and thus includes mostly tests for one's working memory and task-switching requirements. However, only the DSST was included in the processing speed composite score to avoid multicollinearity, as TMT A was additionally used for the calculation of the executive function measure based on the TMT B–A difference score.

**Executive function:** The difference between TMT A and TMT B was used to gain an indicator of executive control abilities (Reitan, 1958; Sánchez-Cubillo et al., 2009).

We applied the tests in the following fixed order: VLMT A, TMT A, TMT B, Corsi A, Corsi B, LNST, DSST, D2 test, VLMT delayed recall (RichtigA7) and MWT-B.

Except for the IQ points based on MWT-B, all test results were raw values. To represent better cognitive performance with higher test results throughout all neurocognitive tests, TMT A and TMT B as well as TMT_A-B were reverse coded.

### 2.4 Psychosocial assessment

To account for additional psychosocial factors potentially influencing cognitive performance, we assessed perceived stress (PSS) and childhood trauma burden (CTQ).

Childhood trauma burden before the age of 18 years was assessed using the German 28-item version of the Childhood Trauma Questionnaire (CTQ) (Wingenfeld et al., 2010), a retrospective self-report instrument assessing emotional, physical and sexual abuse, as well as emotional and physical neglect. Total CTQ scores can be used to reflect overall childhood maltreatment burden.

Perceived stress was assessed using the Perceived Stress Scale (PSS) (Cohen et al., 1983), a self-report questionnaire measuring subjective stress perception.

### 2.5 Statistical analyses

RStudio version 2026.04.0 was used for all statistical analyses. Statistical threshold was set at p = 0.05.

Since neurocognitive test results are strongly influenced by one's education, we controlled for participants' educational level in the following analyses. In addition, perceived stress (PSS) and childhood trauma burden (CTQ) were included as covariates to account for potential psychosocial influences on cognitive performance. To account for population stratification and control for genetic heterogeneity within the study sample, we included multidimensional scaling components (C1 - C8) in all analyses including PRS.

For the MANCOVA analyses, Helmert contrasts were applied for categorical variables to facilitate the estimation and interpretation of Type III effects. In the domain-specific regression analyses, sex was included using the default treatment coding implemented in R.

#### 2.5.1 Associations between BMI, PRS, and general cognitive function

To test our hypotheses of BMI*age and PRS*age interactions on overall cognitive performance, we applied multivariate analyses of covariance (MANCOVA) using ten neurocognitive test scores as dependent variables, following procedures from our previous work (Goltermann et al., 2021). Continuous variables were z-standardized.

a. For MANCOVA A, sex was applied as a fixed factor, while age, years of education, PSS, CTQ and BMI were included as continuous covariates. To assess the possible moderating effects of age and sex on the association between BMI and general cognitive function, we analysed the interaction effects of BMI*age and BMI*sex.
b. Subsequently, we aimed to investigate the overall relationship between PRS and general cognitive function. To this end, we applied a similar multivariate analysis of covariance (MANCOVA B), using the covariate PRS instead of BMI. We furthermore added variables controlling for population heterogeneity (C1-C8) as additional covariates in the model. In accordance with our first MANCOVA model, we again tested for interactions between PRS*age and PRS*sex.
c. In a third model (MANCOVA C), as a sensitivity analysis, we repeated the PRS-based multivariate model MANCOVA B including BMI and BMI*age as covariates to examine whether the age-dependent associations between obesity PRS and cognitive performance remained significant beyond the effects of the BMI*age interaction and current body weight.

#### 2.5.2 Domain-specific regression analyses

To assess the contribution of each cognitive domain on the interaction effects of BMI and PRS with age, separate follow-up regression models were calculated for the six cognitive domain scores, including z-standardized BMI or PRS, z-standardized age, z-standardized years of education, z-standardized PSS, z-standardized CTQ, sex and the interaction term with age (BMI*age or PRS*age) as predictors. PRS models additionally included eight ancestry principal components (C1–C8) to control for population stratification. Details on model diagnostics and sensitivity analyses are provided in the Supplementary Material.

## 3 Results

### 3.1 Associations between BMI, PRS and general cognitive function

a. In MANCOVA A, the interaction between BMI and age was significant (ηp^2^ (BMI*age) = 0.038, F (10, 771) = 3.06, p = 0.001, V = 0.038), indicating that the association between BMI and cognitive performance differed across age. Pillai's trace revealed no significant multivariate main effect of BMI on cognitive performance (ηp^2^ (BMI) = 0.019, F (10, 771) = 1.52, p = 0.128, V = 0.019). In contrast, significant multivariate effects were observed for age (ηp^2^ (age) = 0.379, F (10, 771) = 46.98, p &lt; 0.001, V = 0.379), sex (ηp^2^ (sex) = 0.138, F (10, 771) = 12.32, p &lt; 0.001, V = 0.138), years of education (ηp^2^ (years of education) = 0.110, F (10, 771) = 9.52, p &lt; 0.001, V = 0.110), and childhood trauma burden (ηp^2^ (CTQ) = 0.031, F (10, 771) = 2.45, p = 0.007, V = 0.031). No significant multivariate effect of perceived stress was observed (ηp^2^ (PSS) = 0.009, F (10, 771) = 0.68, p = 0.745, V = 0.009). The BMI*sex interaction was not significant (ηp^2^ (BMI*sex) = 0.015, F (10, 771) = 1.20, p = 0.287, V = 0.015).
b. In MANCOVA B, the interaction between PRS and age was significant (ηp^2^ (PRS*age) = 0.028, F (10, 763) = 2.20, p = 0.016, V = 0.028), indicating that the association between PRS and cognitive performance differed across age. Pillai's trace revealed no significant multivariate main effect of PRS on cognitive performance (ηp^2^ (PRS) = 0.007, F (10, 763) = 0.57, p = 0.839, V = 0.007). In contrast, significant multivariate effects were observed for age (ηp^2^ (age) = 0.404, F (10, 763) = 51.70, p &lt; 0.001, V = 0.404), sex (ηp^2^ (sex) = 0.152, F (10, 763) = 13.66, p &lt; 0.001, V = 0.152), years of education (ηp^2^ (years of education) = 0.111, F (10, 763) = 9.50, p &lt; 0.001, V = 0.111), and childhood trauma burden (ηp^2^ (CTQ) = 0.029, F (10, 763) = 2.27, p = 0.013, V = 0.029). No significant multivariate effect of perceived stress was observed (ηp^2^ (PSS) = 0.009, F (10, 763) = 0.71, p = 0.713, V = 0.009). The PRS*sex interaction was not significant (ηp^2^ (PRS*sex) = 0.006, F (10, 763) = 0.47, p = 0.912, V = 0.006).
c. In the BMI-adjusted sensitivity analysis (MANCOVA C), the PRS*age interaction remained significantly associated with overall cognitive performance after additional adjustment for BMI, the BMI*age interaction, perceived stress, and childhood trauma burden (ηp^2^ = 0.026, F (10, 761) = 2.03, p = 0.028, V = 0.026).

### 3.2 Domain-specific regression analyses

a. Regression analyses of the cognitive domain scores indicated BMI*age effects for episodic memory (B = −0.087, SE = 0.029, p = 0.003) and crystallized intelligence (B = 0.089, SE = 0.032, p = 0.005), suggesting age-dependent associations between BMI and these cognitive domains.

Specifically, increasing age was associated with a more negative relationship between BMI and episodic memory and a more positive relationship between BMI and crystallized intelligence. Little evidence for BMI*age effects was observed for working memory, attention, processing speed, or executive function (Table 2).

**Table 2:** Linear regression models for cognitive domains (BMI).

Predictor	Working memory			Episodic memory			Attention			Processing Speed			Executive Function			Crystallized Intelligence		
	Β^a^	SE^b^	p	B	SE	p	B	SE	p	B	SE	p	B	SE	p	B	SE	p
BMI	−0.041	0.035	0.245	−0.053	0.032	0.096	−0.048	0.034	0.161	−0.010	0.033	0.750	0.061	0.037	0.101	−0.023	0.035	0.511
Age	−0.321	0.036	<0.001	−0.334	0.033	<0.001	−0.415	0.035	<0.001	−0.434	0.033	<0.001	−0.268	0.038	<0.001	0.316	0.036	<0.001
BMI*age	0.017	0.032	0.602	−0.087	0.029	0.003	0.019	0.031	0.546	−0.038	0.030	0.199	0.056	0.034	0.096	0.089	0.032	0.005
Sex	−0.212	0.069	0.002	0.349	0.063	<0.001	0.124	0.067	0.064	0.408	0.064	<0.001	0.011	0.073	0.875	−0.088	0.069	0.198
Years of education	0.173	0.033	<0.001	0.175	0.030	<0.001	0.160	0.032	<0.001	0.159	0.031	<0.001	0.131	0.035	<0.001	0.259	0.033	<0.001
PSS	−0.042	0.035	0.228	−0.030	0.032	0.345	−0.034	0.034	0.320	−0.048	0.032	0.141	−0.081	0.037	0.027	−0.043	0.035	0.216
CTQ	−0.096	0.035	0.006	0.021	0.032	0.508	−0.070	0.034	0.041	−0.095	0.033	0.004	0.006	0.037	0.876	−0.015	0.035	0.674

Note.

^3^p = p-value.

a B = unstandardized regression coefficient.

b SE = standard error.

**Table 3:** Linear regression models for cognitive domains (PRS).

Predictor	Working memory			Episodic memory			Attention			Processing Speed			Executive Function			Crystallized Intelligence		
	Β^a^	SE^b^	p	B	SE	p	B	SE	p	B	SE	p	B	SE	p	B	SE	p
PRS	−0.032	0.033	0.330	−0.015	0.030	0.624	−0.026	0.032	0.419	−0.006	0.030	0.841	0.022	0.035	0.523	0.016	0.033	0.631
Age	−0.323	0.035	<0.001	−0.373	0.032	<0.001	−0.417	0.033	<0.001	−0.427	0.032	<0.001	−0.232	0.036	<0.001	0.317	0.034	<0.001
PRS*age	0.073	0.034	0.032	−0.083	0.031	0.008	0.004	0.033	0.915	0.007	0.032	0.829	0.018	0.036	0.610	−0.025	0.034	0.454
Sex	−0.202	0.069	0.003	0.364	0.063	<0.001	0.131	0.066	0.047	0.401	0.063	<0.001	−0.016	0.072	0.825	−0.093	0.068	0.173
Years of education	0.175	0.033	<0.001	0.187	0.030	<0.001	0.153	0.032	<0.001	0.155	0.031	<0.001	0.114	0.035	0.001	0.252	0.033	<0.001
PSS	−0.045	0.035	0.200	−0.031	0.032	0.330	−0.036	0.034	0.286	−0.052	0.032	0.108	−0.082	0.037	0.027	−0.041	0.035	0.246
CTQ	−0.100	0.036	0.005	0.026	0.033	0.428	−0.074	0.034	0.032	−0.093	0.033	0.005	−0.010	0.038	0.786	−0.022	0.035	0.529

Note.

All models additionally included multidimensional scaling components (C1-8).

^3^p = p-value.

a B = unstandardized regression coefficient.

b SE = standard error.

Years of education showed positive associations across all cognitive domains. Associations of sex, perceived stress, and childhood trauma burden were observed for selected cognitive domains.

**Fig. 1:**
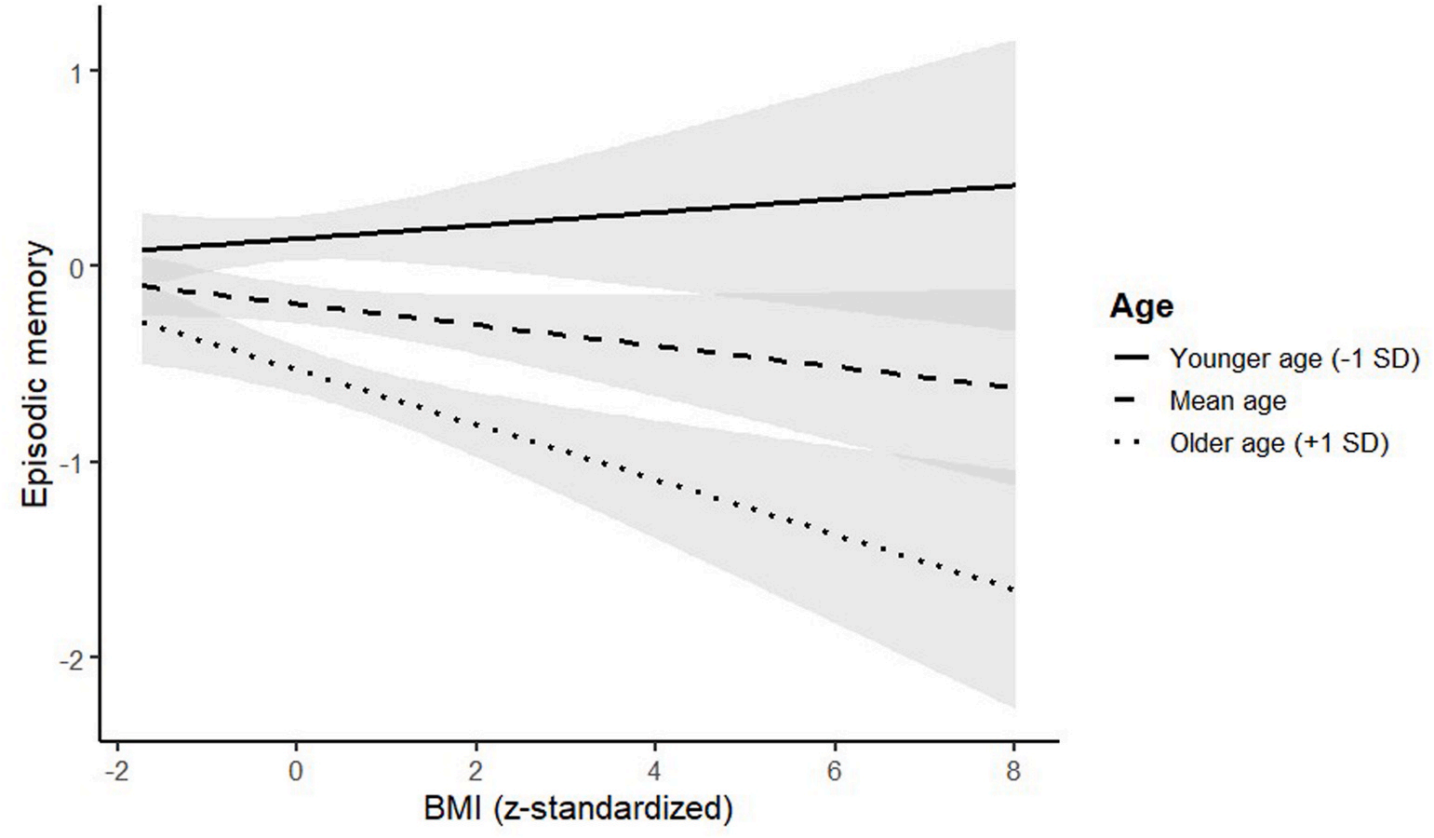
Interaction between BMI and age predicting episodic memory performance. Higher BMI was associated with poorer episodic memory performance at higher age, whereas little association was observed in younger participants. Lines represent predicted values at −1 SD, mean, and +1 SD of age. Shaded areas indicate 95% confidence intervals.

Detailed results are displayed in Table 2 and visualized in Fig. 1.

b. For obesity PRS, regression analyses indicated PRS*age effects for episodic memory (B = −0.083, SE = 0.031, p = 0.008) and working memory (B = 0.073, SE = 0.034, p = 0.032), suggesting age-dependent associations between obesity-related genetic risk and these cognitive domains. Specifically, increasing age strengthened the negative association between obesity-related genetic risk and episodic memory, whereas it strengthened the positive association between obesity-related genetic risk and working memory. Little evidence for PRS*age effects was observed for attention, processing speed, executive function, or crystallized intelligence (Table 3).

Years of education showed positive associations across all cognitive domains. Additional associations of sex, perceived stress, and childhood trauma burden with selected cognitive domains were observed.

**Fig. 2:**
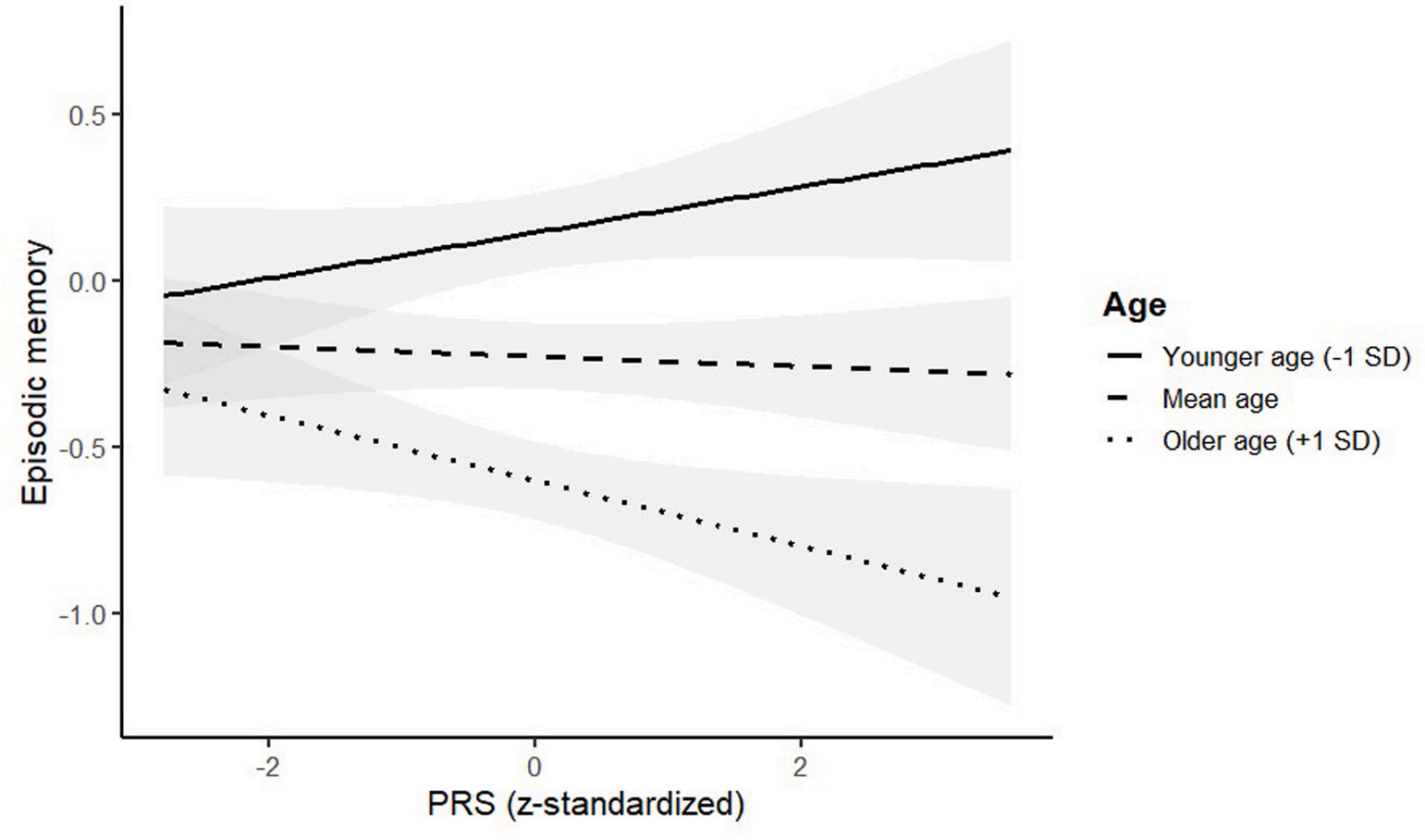
Interaction between PRS and age predicting episodic memory performance. Higher PRS was associated with poorer episodic memory performance at higher age, whereas little association was observed in younger participants. Lines represent predicted values at −1 SD, mean, and +1 SD of age. Shaded areas indicate 95% confidence intervals.

Detailed results are displayed in Table 3 and visualized in Fig. 2.

## 4 Discussion

### 4.1 Key findings

Our study revealed that the associations between both BMI and PRS with cognitive performance were significantly moderated by age. While neither BMI nor PRS showed significant main effects on overall cognitive function, both exhibited age-dependent associations with the multivariate cognitive profile. Notably, the PRS*age interaction remained significant after additional adjustment for BMI and the BMI*age interaction, suggesting that genetic liability for obesity may influence cognitive functioning independently of current body weight. Follow-up analyses indicated that these age-dependent effects were primarily driven by episodic memory, with additional domain-specific associations observed for crystallized intelligence and working memory.

### 4.2 Association between BMI and cognition

Although previous research has repeatedly associated obesity with cognitive impairment (Bischof and Park, 2015; Prickett et al., 2015), we did not observe a significant overall BMI effect in the present analyses. This may be due to the age-dependent nature of the relationship between BMI and cognition, as indicated by the significant BMI*age interaction. This interaction may reduce the average BMI effect observed across the entire sample. Additionally, obesity-related cognitive alterations may heterogeneous, affecting certain cognitive functions rather than overall cognitive functioning. This could limit the detection of a significant multivariate omnibus effect.

Interestingly, our finding of a significant BMI*age interaction in the absence of a significant overall multivariate BMI main effect is in line with previous evidence suggesting that the association between adiposity and cognitive functioning changes across the lifespan (Bischof and Park, 2015). Several studies have reported that obesity during midlife is associated with an increased risk of later cognitive decline (Dahl et al., 2010), whereas associations in older populations appear more heterogeneous, highlighting age as an important moderator of the obesity–cognition relationship (Anstey et al., 2011).

Obesity is characterized by chronic low-grade inflammation, metabolic dysregulation and vascular alterations that have been linked to cognitive decline, increased dementia risk and impaired brain health (Le Thuc and García-Cáceres, 2024; Spyridaki et al., 2016; Livingston et al., 2020). In particular, obesity is associated with endothelial dysfunction, arterial stiffness and impaired cerebrovascular reactivity, which may compromise cerebral blood flow and reduce the delivery of oxygen and nutrients to brain tissue (Balasubramanian et al., 2021). Over time, these vascular alterations may contribute to microvascular damage and structural brain changes that have been associated with poorer cognitive performance (Nguyen et al., 2014; Buie et al., 2019). In addition, neuroimaging studies have reported obesity-related structural and functional brain alterations, including reduced grey matter volume and altered prefrontal functioning (Pannacciulli et al., 2006; Raji et al., 2010; Bobb et al., 2014; Janowitz et al., 2015; Taki et al., 2008; Nyberg et al., 2020).

These obesity-related biological changes may accumulate over time and interact with age-related neurobiological vulnerability, thereby contributing to the stronger BMI–cognition association observed in older individuals. Furthermore, age-related reductions in cognitive reserve may limit compensatory mechanisms, rendering the cognitive consequences of obesity increasingly apparent with advancing age.

Notably, the observed age-dependent association in this study was most pronounced for episodic memory, with higher BMI being associated with poorer episodic memory performance at older ages. Episodic memory has repeatedly been linked to obesity-related cognitive alterations (Cheke et al., 2016). Previous studies suggest that obesity may affect neural systems supporting episodic memory, including the hippocampus and prefrontal cortex, both of which play key roles in memory encoding and retrieval (Kharabian et al., 2016; Cheke et al., 2017).

In contrast, a positive BMI*age interaction was observed for crystallized intelligence. In contrast to episodic memory, crystallized intelligence reflects accumulated knowledge and vocabulary and is generally considered relatively resilient to age-related cognitive decline (Tucker-Drob et al., 2022). This contrasting pattern across cognitive domains may therefore suggest that obesity-related cognitive effects are more likely to affect fluid cognitive processes than well-established knowledge-based abilities.

### 4.3 Association between PRS and cognition

In the present study, no significant overall main effect of obesity PRS on cognitive performance was observed. The absence of a significant overall PRS main effect is consistent with some of the heterogeneous findings reported in the literature. While some studies have demonstrated an association between obesity-related genetic risk and cognitive performance or brain structure (Marioni et al., 2016; Xian et al., 2020; Hagenaars et al., 2016), others have reported only weak or non-significant effects after accounting for relevant covariates (Xian et al., 2020). In view of the modest effect sizes typically observed for polygenic scores, it is conceivable that obesity-related genetic risk influences cognition is only exerted under specific conditions or at particular stages of life.

A salient PRS*age interaction was identified at the multivariate level, indicating that the association between genetic liability for obesity and cognition differs across the lifespan. In this context, Lindenberger and colleagues introduced the resource-modulation hypothesis, which suggests that the decline in anatomical and neurochemical brain resources during the natural aging process modifies the influence of common genetic variations on cognitive function (Lindenberger et al., 2008). Another hypothesis that may explain the weaker manifestation of genetic influences on cognition in younger adults is gene–environment interaction. Beneficial lifestyle factors, including physical activity (Ferencz et al., 2014), a healthy diet (Whalley et al., 2008) and cognitive engagement (Wirth et al., 2014) may buffer or compensate for the adverse effects of genetic risk variants, thereby attenuating their phenotypic expression. The reverse of this effect is also evident, since higher blood pressure (Frias et al., 2014) and increased cardiovascular risk (Raz et al., 2009) have been shown to interact with risk alleles resulting in negative effects on cognition. Besides that, genetic factors can be enhanced through gene-environment correlations over time, as individuals tend to select experiences in line with their genetic predispositions, thereby enhancing genetic variations (Kendler and Eaves, 1986).

Notably, the PRS*age interaction remained significant following adjustment for BMI and the BMI*age interaction. This suggests that obesity-related genetic liability may influence cognitive function through mechanisms beyond current body weight, in line with recent work by Saeed and colleagues highlighting that many obesity-associated genetic variants act through central nervous system pathways and may influence both adiposity and brain function (Saeed et al., 2025).

Indeed, up to 75% of obesity-related genes express preferentially in the brain (Locke et al., 2015; Vainik et al., 2018) and neuroimaging research shows associations between a higher polygenic risk score for obesity and changes in brain structure, such as a reduced occipital surface area (Opel et al., 2020b). Furthermore, Morys and colleagues reported associations between obesity PRS, the neuroanatomy of frontal and temporal brain regions, and impulsivity, supporting the notion that obesity-related genetic liability may affect neural systems relevant for cognitive functioning (Morys et al., 2023). Alternatively, obesity PRS may capture the cumulative effects of lifelong adiposity-related risk, which may not be fully reflected by BMI as a cross-sectional measure of current body weight.

Follow-up analyses revealed that the age-dependent association between obesity PRS and cognition was most pronounced for episodic memory and working memory. Specifically, the negative association between obesity-related genetic risk and episodic memory performance became stronger with increasing age, whereas a positive age-dependent association was observed for working memory. The episodic memory finding is particularly noteworthy, as previous research has linked obesity to alterations in hippocampal and medial temporal lobe structures that support episodic memory (Raji et al., 2010; Hayes et al., 2020). The fact that the PRS*age interaction emerged most clearly in this domain may therefore suggest a selective sensitivity of memory-related neural systems to obesity-related genetic liability. In contrast, the positive age-dependent association observed for working memory should be interpreted cautiously, given the limited prior evidence and lack of a clear mechanistic explanation. Notably, episodic memory also emerged as the domain most strongly associated with the BMI*age interaction, lending additional support to the robustness and potential relevance of the episodic memory findings.

### 4.4 Potential influences of sex, education, stress and trauma

In addition to age, several demographic and psychosocial factors may influence the complex relationships among obesity, cognition, and genetic liability.

In contrast to the clear moderating effect of age on the associations between BMI/PRS and cognition, no evidence was found for a moderating role of sex. While significant sex differences were observed in several cognitive domains, these effects reflected differences in overall performance rather than differential associations between BMI/PRS and cognition. This is notable given previous reports of sex-specific effects of obesity and genetic factors on cognitive functioning (Opel et al., 2020b; Gustafson, 2012; Milanova et al., 2021; Karlsson et al., 2021; Ratnu et al., 2017). However, our finding of the PRS affecting cognition in a consistent manner across different sexes is an important addendum to the previous study by Xian et al., that elaborated on the association between PRS and cognition based on an all-male study sample (Xian et al., 2020). The present findings suggest that the association between obesity-related genetic liability and cognition is largely comparable across sexes, although further research is needed to clarify the role of sex in obesity–cognition relationships.

For instance, higher childhood trauma scores were associated with poorer performance in working memory, attention and processing speed. These findings are consistent with previous evidence suggesting that early-life adversity may have lasting effects on neurocognitive systems involved in attentional control, information processing and working memory, potentially through long-term alterations in stress-related neurobiological pathways (Gould et al., 2012; Teicher and Samson, 2016).

In contrast, perceived stress showed a more selective association with cognition and was only related to executive functioning. This is consistent with prior evidence indicating that stress-related cognitive effects are particularly pronounced in prefrontal cortex-dependent processes such as cognitive control and executive functioning (Arnsten, 2009).

As expected, higher educational attainment was consistently associated with better cognitive performance across all cognitive domains, in line with evidence suggesting that education contributes to cognitive reserve and is associated with higher levels of cognitive functioning across the lifespan (Lövdén et al., 2020).

Importantly, the observed BMI*age and PRS*age interactions remained significant after adjustment for sex, years of education, perceived stress and childhood trauma, indicating that age-related differences in the association between obesity-related factors and cognition are unlikely to be attributable solely to psychosocial adversity or educational background.

### 4.5 Strengths and limitations

A strong point of our study lies in the large sample size including women and men of various age groups. Since we applied a very broad neurocognitive test battery for various cognitive domains and always tested for educational and psychosocial bias, we were able to gain a comprehensive understanding of our participants’ cognitive abilities.

A limitation of our work is the cross-sectional design that prevents us from drawing conclusions on causality. It is important to note that domain-specific regression analyses were conducted to characterize significant multivariate findings and should therefore be interpreted as exploratory. Furthermore, experts have criticized the BMI as measurement instrument for obesity as it fails to differentiate between muscle mass and fat (Stevens et al., 2008). Future studies should therefore consider further measures of adiposity such as the waist-to-hip-ratio or body fat percentage. Moreover, there might be other influential factors not considered here that could moderate the association between BMI/PRS and cognition. For instance, physical activity (Dore et al., 2008), sleep (Lo et al., 2016), parity in female participants (Lee et al., 2025), exposure to hormonal medication (Sharma et al., 2023), menopause-related hormonal changes (Conde et al., 2021) and psychiatric or somatic disorders have been associated with cognitive functioning and may therefore represent additional factors relevant to the relationship between obesity and cognition. The restriction to psychiatrically healthy individuals reduced potential confounding by psychiatric illness and psychotropic medication but may limit the generalizability of the findings to clinical populations.

As new and improved PRS calculation methods are now being established (Ge et al., 2019), future studies should verify the results presented here with more up-to-date methods. While the present study focused on the obesity polygenic risk score as a composite measure of genetic liability, future studies may benefit from examining the extent to which the observed associations reflect broader obesity-related genetic architecture versus effects attributable to APOE specifically (Vasiljevic et al., 2023). Furthermore, participants were restricted to individuals of West-European ancestry to reduce potential bias due to population stratification and to maximize the validity of PRS analyses based on a predominantly European-ancestry discovery GWAS. Therefore, the extent to which these findings generalize to other ancestral groups remains unclear and warrants investigation in more diverse populations.

Importantly, our sample was restricted to adults aged 18–65 years. Therefore, the present findings cannot determine whether the observed age-dependent associations continue into late life, where previous studies have reported more heterogeneous obesity–cognition relationships and findings consistent with an obesity paradox (Anstey et al., 2011). In addition, it now appears mandatory to elucidate whether negative effects of BMI and PRS on cognition could be reversed, at least to some extent, by therapeutic interventions such as cognitive training and educational activities. To disentangle the complex relationship between obesity, cognition, and genetics and to evaluate prevention options, it is essential to conduct longitudinal studies covering the entire age spectrum.

## 5 Conclusion

In conclusion, our findings suggest that both BMI and obesity-related genetic liability are associated with age-dependent differences in cognitive functioning, with episodic memory emerging as the cognitive domain most consistently related to both factors. Notably, the age-dependent association between obesity PRS and cognition remained significant after adjustment for BMI and the BMI*age interaction, suggesting that obesity-related genetic liability may influence cognition through pathways beyond current body weight. Together, these findings highlight age as an important moderator of the associations between obesity-related factors and cognition. Future longitudinal studies are needed to clarify the mechanisms underlying these associations, evaluate their temporal dynamics, and determine their potential relevance for strategies aimed at preserving cognitive health across the lifespan.

## CRediT authorship contribution statement

**Mathilda Recht:** Conceptualization, Data curation, Formal analysis, Investigation, Methodology, Validation, Visualization, Writing – original draft, Writing – review & editing. **Kathrin Finke:** Data curation, Writing – review & editing. **Marius Gruber:** Data curation, Writing – review & editing. **Andreas J. Forstner:** Data curation, Writing – review & editing. **Janik Goltermann:** Data curation, Writing – review & editing. **Maike Richter:** Data curation, Writing – review & editing. **Benjamin Straube:** Data curation, Writing – review & editing. **Elisabeth Schrammen:** Data curation, Writing – review & editing. **Moritz Rau:** Data curation, Writing – review & editing. **Jonathan Repple:** Data curation, Writing – review & editing. **Kira Flinkenflügel:** Data curation, Writing – review & editing. **Judith Krieger:** Data curation, Writing – review & editing. **Anna Kraus:** Data curation, Writing – review & editing. **Elisabeth J. Leehr:** Data curation, Writing – review & editing. **Tiana Borgers:** Data curation, Writing – review & editing. **Tilo Kircher:** Data curation, Writing – review & editing. **Udo Dannlowski:** Data curation, Writing – review & editing. **Nils Opel:** Conceptualization, Data curation, Formal analysis, Methodology, Project administration, Supervision, Writing – review & editing.

## Declaration of competing interest

The authors declare that they have no known competing financial interests or personal relationships that could have appeared to influence the work reported in this paper.

## Data Availability

Data will be made available on request.
