# Supplementary material for: Associations between polygenic risk for obesity, body mass index and cognitive performance: Age as moderating factor
Source: Brain Behav Immun Health. 2026 Jul 31;56:101321. doi: 10.1016/j.bbih.2026.101321 (PMC13471357; doi:10.1016/j.bbih.2026.101321)
Supplement: Supplementary file 1 [file mmc1.docx]

# **Supplements**

**Study sample**

Details of the study population are presented in table S1.

**Domain specific regression analyses**

To assess multicollinearity, variance inflation factors (VIFs) were examined for all regression models, including those containing interaction terms. All VIF values are presented in table S2 and were low (all < 2), indicating no evidence of problematic multicollinearity. Visual inspection of Q–Q plots revealed minor deviations from normality, particularly in the tails of the distribution. However, given the large sample size, the regression models were considered robust to moderate violations of the normality assumption. As an additional sensitivity analysis, all regression models were re-estimated using HC3 heteroscedasticity-robust standard errors. The direction and significance of the reported interaction effects remained unchanged, further supporting the robustness of the findings.

# **Tables**

**Table S1**

*Inclusion and exclusion criteria of the FOR2107 study population.^1^*

| Condition | Affective disorders (MDD n = 700; BD n = 300) | Psychotic disorders (SZ n = 200; SZA n = 100) | Healthy subjects without risks (n = 1000) | Healthy subjects, genetic risks (n = 200)^e^ | Healthy subjects, environmental risks (n = 200)^f^ | Healthy subjects, environmental and genetic risks (n = 100)^e,f^ |
| --- | --- | --- | --- | --- | --- | --- |
| Verbal IQ > 80 | + | + | + | + | + | + |
| West-European ancestry | + | + | + | + | + | + |
| MRI compatibility^a^ | + | + | + | + | + | + |
| + substance related disorders | - | -^c^ | - | - | - | - |
| History of severe neurological^b^ or medical disorders^d^ | - | - | - | - | - | - |
| At least one 1^st^ degree relative with lifetime diagnosis MDD or BD | + | + | -^e^ | + | -^e^ | + |
| At least one CTQ subscale reaching the threshold for maltreatment | + | + | -^f^ | -^f^ | + | + |
| Family history of any psychiatric disorder | + | + | - | -^g^ | - | -^g^ |
| Current psychotropic medication | + | + | - | - | - | - |
| Current benzodiazepine use | -^i^ | -^i^ | - | - | - | - |
| Past or present SCID-diagnosis^h^ | + | + | - | - | - | - |

*Note.*

^a^Exclusion due to, e.g., metal implants, pacemakers, claustrophobia, pregnancy, etc.

^b^E.g., seizures, stroke, multiple sclerosis, dementia, traumatic brain injury

^c^In case of SZA and SZ cannabis abuse is tolerated, these patients are included, other comorbid substance-related diagnoses are excluded

^d^E.g., cancer, auto-immune disorders, chronic inflammatory diseases, and cardio-vascular diseases

^e^Subjects with at least one relative with the diagnosis of MDD or BD

^f^At least one subscale of the CTQ reaching the threshold for maltreatment

^g^Include in case of family history (fist degree relative) of MDD or BD is inclusion criterion, otherwise exclude

^h^SCID-confirmed diagnosis of MDD and BD (any polarity); according to DSM-IV criteria (296.xx) or SZ (295.1-3/295.9); and SZA (295.7). Dysthymia and cyclothymia are not inclusion criteria, but are allowed as comorbid disorders within any patient group

^i^Wash-out of > 3 half-lives required to be included (within patients only)

**Table S2**

*Variance inflation factors (VIFs) for predictors included in the BMI and PRS regression models.*

| **Predictor** | **Adjusted GVIF (BMI models)** | **Adjusted GVIF (PRS models)** |
| --- | --- | --- |
| **BMI / PRS** | 1.025 | 1.026 |
| **Age** | 1.025 | 1.026 |
| **Sex** | 1.022 | 1.012 |
| **Years of education** | 1.020 | 1.015 |
| **PSS** | 1.063 | 1.070 |
| **CTQ** | 1.086 | 1.093 |
| **C1-C8** | - | 1.003 – 1.015 |
| **Note.** *VIFs were calculated using a predictor-based approach. Accordingly, interaction terms are not reported separately, as their contribution to multicollinearity is incorporated into the VIF estimates of the corresponding predictors.* | | |

References

1	Kircher T, Wöhr M, Nenadic I, Schwarting R, Schratt G, Alferink J *et al.* Neurobiology of the major psychoses: a translational perspective on brain structure and function-the FOR2107 consortium. *European archives of psychiatry and clinical neuroscience* 2019; 269: 949–962.
